# Modulation of gut microbiota in Graves’ orbitopathy: *Prevotella* dominance and atorvastatin’s impact

**DOI:** 10.1186/s40168-025-02219-2

**Published:** 2025-12-29

**Authors:** Danyu Wang, Yaonan Chen, Junpeng Yang, Yun Zhang, Xinru Deng, Yalei Liu, Yiqi Chen, Xueli Yang, Xiudan Wang, Chenghong Liang, Qinyuan Xie, Yibin Hao, Huijuan Yuan

**Affiliations:** 1 Department of Endocrinology, Henan Provincial Key Medicine Laboratory of Intestinal Microecology and Diabetes, Diabetes Microecology Diagnosis and Treatment and Transformation Engineering Research Center of Henan Province, Henan Provincial People’s Hospital, People’s Hospital of Zhengzhou University, Zhengzhou, 450003 China; 2 Henan Provincial People’s Hospital, Zhengzhou, 450003 China

**Keywords:** Graves’ orbitopathy, Gut microbiota, Atorvastatin, *Prevotella*

## Abstract

**Background:**

The gut microbiota in patients with Graves’ orbitopathy (GO) may influence the disease’s progression, but its specific role and function in the progression of GO treatment are not well understood.

**Methods:**

We performed fecal microbiota sequencing using the 16S rRNA-gene sequencing on patients with GO (*n* = 48), Graves’ disease (GD, *n* = 40), and healthy controls (HC, *n* = 36). Subsequently, fecal samples from patients with GO, GD, and healthy donors were transplanted into antibiotic-treated pseudo-germ-free mice. Finally, the 48 patients with GO were randomly divided into two groups: one group received intravenous glucocorticoids (ivGC) and atorvastatin (*n* = 24), while the other group received ivGC only (*n* = 24), to observe the effects of atorvastatin on GO progression and its impact on gut microbiota.

**Results:**

Patients with GO exhibit a distinct gut microbiota composition, particularly marked by increased levels of *Prevotella* and *Bacteroides*, compared to patients with GD and HC. Correlation analysis revealed a direct positive association between *Prevotella* and thyrotropin receptor antibody levels. Antibiotic-treated pseudo-germ-free mice that received fecal transplants from patients with GO exhibited a slower rate of weight gain, significant impairment of intestinal barrier integrity, and markedly increased levels of serum LBP and inflammatory factors. A combined treatment regimen of ivGCs and atorvastatin significantly reduced ocular clinical symptoms in patients with GO, including clinical activity score, exophthalmos, and intraocular pressure, while also promoting a healthier gut microbiota composition and a reduction in *Prevotella* levels.

**Conclusions:**

Gut microbiota imbalance, particularly involving *Prevotella*, contributes to GO’s development and progression. Atorvastatin may slow GO progression by correcting dysregulated gut microbiota, especially reducing *Prevotella*.

Video Abstract

**Supplementary Information:**

The online version contains supplementary material available at 10.1186/s40168-025-02219-2.

## Introduction

Graves’ orbitopathy (GO), the primary extrathyroidal manifestation of Graves’ disease (GD), is an autoimmune condition triggered by the activation of orbital fibroblasts and thyroid cells by autoantigens [1–3]. The characteristic of this situation is that plasma cells in the bloodstream produce thyroid stimulating hormone receptor antibodies (TRAb), which specifically target the thyroid stimulating hormone receptor [4]. These autoantibodies interact with the thyrotropin receptor (TSHR) in orbital preadipocytes and fibroblasts, triggering lipogenesis and the transformation of fibroblasts into myofibroblasts [1]. Consequently, this activity results in tissue swelling behind the eyes, causing them to bulge and restrict movement. Patients with GO often suffer from noticeable disfigurement, including protruding eyes and a persistent stare, along with signs of inflammation and eye dysfunction [5]. Notable among these symptoms is double vision, which, in severe cases, can lead to loss of vision [5]. Although Teprotumumab has been listed by the Food and Drug Administration (FDA) as a first-line treatment for GO [6], in China, high-dose intravenous glucocorticoids (ivGCs) remain the preferred option for treating moderate to severe active GO due to their cost-effectiveness [7]. However, the specific mechanisms by which ivGCs treat GO are still not fully understood.

Mounting evidence increasingly supports the pivotal role of gut microbiota in the onset and progression of autoimmune diseases, notably GD and GO [8–10]. Research by Biscarini et al. has identified disturbances within the gut microbiome of patients with GD and GO, linked to levels of TRAb [10]. Further insights from the MicroGO cohort study have underscored a marked rise in intestinal barrier permeability among patients with GO, correlating with intensified inflammation and the aggregation of myofibroblasts in GO [9]. Interventions in animal models have validated the influence of the gut microbiota on TSHR-mediated GO [8], yet the causal relationship between the gut microbiome and GO, as well as its role during treatment, remains unclear.

Recent studies have shown that combined treatment with ivGCs and atorvastatin significantly improves outcomes for patients with moderate to severe GO [11]. Vieira-Silva et al. has revealed that the disruption of microbiota linked to obesity exhibits a negative correlation with statin therapy [12]. A study utilizing the MetaCardis cohort for a population-based cross-sectional examination of the gut microbiome suggests that statin therapy may promote a shift in the gut microbiota towards a healthier composition, evidenced by notable changes in the prevalence of taxa like *Prevotella* and *Bacteroides* [13]. Furthermore, these modifications in the gut microbiome composition could influence how an individual responds to statin treatment [14].

However, the potential benefits of statin drugs in modulating microbiota have yet to be further assessed in prospective clinical trials, including those involving GO, thus it remains uncertain whether their effects can be replicated in a random population. This study first examines the potential association between GO and a distinct gut microbiota composition through a case–control study involving GO, GD, and HC groups. It then investigates the potential causal role of the gut microbiota in GO progression by transplanting fecal samples from these groups into antibiotic-treated, pseudo-germ-free BALB/c mice. Finally, an interventional study assesses whether atorvastatin, in combination with ivGCs, can alleviate the progression of GO by modulating the gut microbiota.

## Materials and methods

We designed and performed two prospective clinical studies, including a case–control study and an interventional study, as well as an animal study.

### Clinical study

Both the case–control and intervention studies received approval from the Medical Ethics Committee of Henan Provincial People's Hospital (Eth.202218) and all subjects provided written informed consent. Additionally, the intervention study was registered with the Chinese Clinical Trial Registry (ChiCTR2200060406) and carried out from May 2022 to May 2023.

### Case–control study

#### Study subjects

Between May 2022 and May 2023, patients diagnosed with GO (GO group, *n* = 48) and patients diagnosed with GD (GD group, *n* = 40) were enrolled from the Endocrinology and Ophthalmology Departments at Henan Provincial People’s Hospital. Additionally, healthy controls (HC group, *n* = 36) were recruited through the hospital’s Health Management Center. All three groups of subjects were matched according to gender and age.

#### Inclusion criteria

The inclusion criteria for GO group were as follows: (1) An age range between 18 and 65 years; (2) a confirmed diagnosis of GO as per the diagnostic guidelines of European Group on Graves'Orbitopathy (EUGOGO), characterized by eyelid retraction accompanied by one or more of the following: abnormal thyroid function, exophthalmos, optic nerve dysfunction, or extraocular muscle involvement. In cases lacking eyelid retraction, the presence of abnormal thyroid function is mandatory, along with at least one of the aforementioned conditions. (3) Patients exhibiting moderate-to-severe and active GO, evidenced by a clinical activity score (CAS) ≥ 3, in alignment with the European Group on Graves'Orbitopathy (EUGOGO) recommendations [7].

The inclusion criteria for the GD group were refined as follows: (1) Patients aged between 18 and 65 years; (2) diagnosis in accordance with GD standards (as defined by the diagnostic guidelines of American Thyroid Association (ATA) [15]); and (3) absence of GO symptoms.

#### Exclusion criteria

The exclusion criteria applicable to all three groups included (1) consumption of antibiotics, probiotics, glucocorticoids, or selenium supplements within the past three months; (2) prior orbital decompression surgery for GO; (3) a history of eye trauma or any ocular conditions other than GO; (4) significant complications associated with GD, such as thyroid storm; (5) presence of diabetes, severe organic illnesses, or autoimmune disorders; (6) moderate to severe renal dysfunction (serum creatinine levels exceeding 2 mg/dL) or significant liver function abnormalities (alanine aminotransferase (ALT), aspartate aminotransferase (AST) levels more than double the normal upper limit); (7) infectious conditions like tuberculosis or HIV; (8) history of surgeries or disorders pertaining to the digestive system; (9) excessive alcohol consumption (defined as drinking on more than five occasions per week, consuming over 100 g of distilled spirits, more than 250 g of yellow wine, or over five bottles of beer); and (10) women during pregnancy or lactation periods.

#### Sample collection

Stool, blood, and ocular surface microbiome from all subjects were collected by the same technician after an 8-h fasting period. The stool and ocular surface microbiome samples were promptly stored in a freezer at − 80 °C within ten minutes of collection until use. Meanwhile, the blood samples were transported to the Laboratory Department of Henan Provincial People’s Hospital within 30 min for thyroid function and thyroid autoantibody testing.

#### Clinical data collection

All participants were evaluated for CAS, proptosis, ocular motility restriction, diplopia, and intraocular pressure. Demographic and health-related data, including name, gender, age, medication, surgical, and past medical history, were gathered using a detailed questionnaire. Measurements of weight and height were conducted for all subjects early in the morning on an empty stomach, with everyone wearing uniform and lightweight clothing. Serum levels of total cholesterol (TC), triglycerides (TG), low-density lipoprotein cholesterol (LDL-C), and high-density lipoprotein cholesterol (HDL-C) were measured in all participants through clinical laboratory testing. Serum levels of thyroid-stimulating hormone (TSH), free triiodothyronine (FT3), free thyroxine (FT4), and TRAb were determined using the Cobas e602 analyzer (Roche Diagnostics, Basel, Switzerland). Furthermore, levels of serum thyroglobulin antibody (TgAb) and thyroid peroxidase antibody (TPOAb) were measured using the UniCel DxI 800 analyzer (Beckman Coulter, Inc., USA).

### Intervention study

#### Study subjects

The patients with GO (*n* = 48) enrolled in our intervention study were derived from our case–control study and had not undergone treatment with atorvastatin or ivGCs therapy. These patients were randomly allocated into two groups for a 12-week intervention. The treatment regimen for one group involved a combination of ivGCs and atorvastatin (ST group, *n* = 24), whereas the other group was treated exclusively with ivGCs (NST group, *n* = 24).

#### Intervention procedures

In accordance with the EUGOGO guideline protocol [7], both cohorts commenced with baseline intravenous methylprednisolone (Pfizer Inc., USA) injections, initially at a dose of 500 mg weekly for the first six weeks, then reducing to 250 mg weekly for the subsequent 6 weeks, achieving a total cumulative dose of 4.5 g. To mitigate potential ivGC-induced side effects, all subjects received daily omeprazole (40 mg, AstraZeneca, UK). Furthermore, the ST group was also prescribed a daily oral dose of 20 mg atorvastatin (Pfizer Inc., USA) throughout the 12-week period.

Patients with GO receive weekly ivGCs treatments at the Endocrinology Department of the Henan Provincial People’s Hospital. During each outpatient clinic visit, we systematically inquire about any adverse reactions the patients may have encountered. This encompasses all undesirable symptoms or signs that have emerged following the initiation of treatment. We also perform targeted assessments of safety biochemical indicators to monitor for potential side effects. All patients who were randomly assigned to one of the treatment groups reported adverse events, and these were documented and coded according to the Medical Dictionary for Regulatory Activities standards recommended by the International Conference on Harmonisation of Technical Requirements for Registration of Pharmaceuticals for Human Use [16]. At the conclusion of the 12th week, patients are subjected to a CAS assessment and a thorough evaluation of thyroid-related parameters, employing the methodologies established at the outset of treatment. Additionally, at the end of the 12 weeks, we also collect the patients’ stool samples.

#### Study outcomes

The primary outcome metrics focused on the alterations in the CAS and the composition of the gut microbiota by the 12th week. The secondary outcomes pertained to modifications in additional clinical parameters.

### Animal study with BALB/c mice

The animal study’s experimental procedures were approved by the Committee of the Animal Experimental Center at Zhengzhou University (ZZU-LAC2023032410), adhering strictly to the established guidelines of the committee.

### Preparation for fecal microbiota transplantation

#### Antibiotic treatment

Five-week-old BALB/c mice (*n* = 36) were maintained at the Zhengzhou University Experimental Animal Center under SPF conditions. They were provided with sterile standard feed, and the room was kept at a temperature of 22 ± 2 °C with 50 ± 10% humidity, following a 12-h light/dark cycle with lights turning on at 7 AM. After a week-long acclimatization period, all the BALB/c mice received drinking water supplemented with a cocktail of broad-spectrum antibiotics—vancomycin (0.5 g/L), streptomycin sulfate (1 g/L), ampicillin (1 g/L), and metronidazole (1 g/L)—for 3 days to eliminate their gut microbiota.

#### Preparation of transplants

The transplants were prepared exclusively in an anaerobic chamber (80% N_2_, 10% CO_2_, and 10% H_2_, Don Whitley Scientific, UK). Equal volumes of frozen fecal samples from individuals with GO (who had not received atorvastatin or ivGCs therapy) (*n* = 5), those with GD (*n* = 5), and HC (*n* = 5) were carefully thawed and then combined at a controlled temperature of 37 °C. Each sample of the mixed fecal material, weighing 1 g, was suspended in 50 mL of a sterile Ringer’s solution [9 g/L sodium chloride, 0.4 g/L potassium chloride, 0.25 g/L calcium chloride dihydrate, 0.05% (W/V) l-cysteine hydrochloride]. This suspension was subjected to vigorous mixing using a vortex for 5 min, followed by a settling period of 5 min to allow the solid particles to settle by gravity. The resultant clear supernatant was carefully decanted into a sterile tube. To this, an equivalent volume of 20% (W/V) skim milk was added, forming the transplantation medium. This mixture was freshly prepared on the day of the transplantation experiment, with any excess being preserved at − 80 °C for future inoculations.

### Fecal microbiota transplantation in BALB/c mice

BALB/c mice were randomly divided into three groups: mGO (*n* = 12), mGD (*n* = 12), and mHC (*n* = 12). These groups received oral gavage with fecal microbiota transplants sourced from donors with GO (who had not received atorvastatin or ivGCs therapy), GD, and HC, respectively. The gavage was administered on days 1, 3, 5, 9, and 13, resulting in a total of five sessions.

Health monitoring of the mice was conducted weekly, with particular attention to changes in body weight, diet, and water consumption. On day 15, the mice were euthanized, and their colon tissues were harvested for zonula occludens-1 (ZO-1) and Occludin protein level analysis.

Blood samples were harvested from the tail vein at two distinct junctures: subsequent to antibiotic treatment but prior to the initial gavage, and once more on the 15th day, aimed at evaluating markers of inflammation. These samples were then allowed a resting period of 30 min before undergoing centrifugation at 1000 g for 30 min. Following this process, the supernatant was carefully collected and securely stored at − 80 °C until required for analysis.

### Immunofluorescence analysis

To explore the disruption of the intestinal barrier, we performed protein expression level detection of the intestinal tight junction proteins ZO-1 and Occludin. The procedure began with dewaxing colon paraffin sections and washing them in distilled water, followed by antigen retrieval in EDTA (pH 8.0), microwaved first at medium for 9 min, rested for 8, and then at medium–low for 7 min. Primary antibodies ZO-1 and Occludin were applied at dilutions of 1:5000 and 1:500, respectively, followed by horseradish peroxidase (HRP)-conjugated and cyanine 3 (CY3)-conjugated goat anti-rabbit IgG as secondary antibodies. BSA blocked serum for 30 min before the primary antibody was applied and overnight incubation at 4 °C in a humid chamber occurred. The slide then received the HRP-conjugated secondary antibody for 50 min at room temperature, and tyramide signal amplification (TSA) dye was applied. After microwave treatment, a second round of primary and secondary antibodies was applied. 4′,6-Diamidino-2-phenylindole (DAPI) counterstained nuclei, with a 10-min dark incubation, followed by autofluorescence quencher solution B for 5 min. The slide was then washed for 10 min, mounted, and imaged.

### ELISA analysis

Commercially available ELISA kits were utilized to measure the levels of inflammatory factors in serum and small intestine tissue, tumor necrosis factor-α (TNF-α) (GEM0004, Servicebio, Wuhan, China; E-MSEL-M0002, Elabscience, Wuhan, China), interleukin-1β (IL-1β) (GEM0002, Servicebio, Wuhan, China; E-MSEL-M0003, Elabscience, Wuhan, China), interleukin-6 (IL-6) (GEM0001, Servicebio, Wuhan, China), interleukin-17 (IL-17) (EK217/2, Multi Sciences, Hangzhou, China; E-MSEL-M0006, Elabscience, Wuhan, China), transforming growth factor-β (TGF-β) (EK981-24, Multi Sciences, Hangzhou, China; E-EL-0162, Elabscience, Wuhan, China), and lipopolysaccharide (LPS)-binding protein (LBP) (E-EL-M2686, Elabscience, Wuhan, China).

### Gut microbiota analysis

#### DNA extraction and 16S rRNA gene V3-V4 region sequencing

Genomic DNA was extracted from fecal samples utilizing the E.Z.N.A.® Stool DNA Kit (Omega Bio-tek, Inc., GA). To amplify the V3–V4 region of the 16S rRNA gene, the forward primers 341 F (5′-CCTACGGNGGCWGCAG-3′) and the reverse primer 805R (5′-GACTACHVGGGTATCTAATCC-3′) were employed. Following amplification, sequencing of the resulting amplicons was performed on the MiSeq platform (Illumina Inc., USA).

#### Sequencing data analysis

Raw sequencing data were processed with Cutadapt (v1.18) to remove primer/adapter sequences. Demultiplexed paired-end reads were subsequently imported into QIIME2 (version 2024.2, http://qiime2.org/) [17] for downstream analysis. Denoising was conducted using the DADA2 algorithm [18], optimizing the process by truncating forward and reverse reads at positions 277 and 217, respectively, to remove low-quality regions. Since primers had already been removed, no further trimming was applied to the read ends. Additionally, chimeric sequences were filtered by ensuring that candidate amplicon sequence variants (ASVs) matched or exceeded the abundance of their inferred parent sequences. ASVs were classified taxonomically using the SILVA 138.2 (http://www.arb-silva.de) [19] reference database via a pre-trained Naïve Bayes classifier, assigning hierarchical taxonomic labels (e.g., phylum to genus).

To ensure data robustness, a multi-step filtering strategy was employed. This included the removal of low-confidence ASVs, where features occurring fewer than 2 times across all samples or appearing in fewer than 2 biological replicates were discarded. Contaminants were excluded by systematically removing ASVs annotated as mitochondrial, chloroplast, or archaeal sequences. Additionally, samples with fewer than 4000 total reads were excluded to avoid biases from insufficient sequencing depth. To account for uneven sequencing depth across samples, a rarefaction-based normalization was applied. The filtered feature table was subsampled without replacement for 100 iterations, with each iteration drawing 10,000 sequences per sample. This process generated a consensus normalized table where the total sequence count per sample was standardized to 1,000,000 reads, ensuring comparability in downstream ecological analyses.

Alpha diversity analysis reflecting microbial community richness and diversity were performed using Mothur (version 1.42.1) [20], where Shannon and Simpson indices indicate microbial diversity, and abundance-based coverage estimator (ACE) and Chao indices reflect microbial richness. We utilized QIIME 2 for calculating the β-diversity based on Bray–Curtis distance matrix, and employed the vegan package within R (version 3.6.0, https://www.r-project.org/) to conduct Bray–Curtis based principal coordinate analysis (PCoA) analysis and visualization. Significance of the partitioning was assessed using PerMANOVA analysis. Furthermore, the linear discriminant analysis effect size (LEfSe) method (version 1.1, https://github.com/SegataLab/lefse) [21], which is grounded in linear discriminant analysis (LDA) effect size, was applied to pinpoint communities or species significantly influencing sample partitioning. We conducted MaAsLin2 analysis to explore the correlation between different abundant bacterial genera and a range of clinical parameters (including age, medications, and disease duration). Specifically, we conducted correlation analysis between the gut microbiota and thyroid-related clinical parameters, such as FT3, FT4, TRAb, TPOAb, TgAb, and TSH, using *Spearman* analysis. Furthermore, *Pearson* analysis was applied to explore the correlation between *Prevotella* and TRAb.

The sequencing and analysis of the ocular surface microbiota were similar to those of the gut microbiota. Additionally, we performed a heatmap analysis to examine the differential gut microbiota identified in the GO group and its presence in the ocular surface microbiota across the GO, GD, and HC groups.

### Statistical analysis

Statistical analysis was performed using R (version 3.6.0). The clinical and animal data**'**s continuous variables were assessed for normality using the Shapiro–Wilk test. For normally distributed continuous data, mean ± standard deviation (SD) was reported, and one-way ANOVA test was used to compare the three groups in the case–control study. Post hoc analysis was conducted with the least significant difference (LSD) *t* test. In intervention studies, paired *t* test was used for within-group comparisons and independent sample *t* test for between-group comparisons.

Non-normally distributed clinical and animal data variables and gut microbiome data were expressed as the median (interquartile range). These data were compared across the three groups in the cross-sectional study using the Kruskal–Wallis test. Post hoc analysis was done with the Mann–Whitney* U* test with Bonferroni correction. Wilcoxon test was used for within-group comparisons in intervention studies and Mann–Whitney* U* test for between-group comparisons.

Categorical data were reported as frequencies (%) and analyzed with Chi-square test. A *p* value < 0.05 was considered statistically significant.

### Sample size estimation

Based on the changes in CAS observed in our study, we set α = 0.05 and 1-β = 0.9 (power), estimated a 10% loss to follow-up, and calculated that approximately 42 subjects would be required. The sample size calculation was based on a margin of error of 0.875, a common standard deviation of 0.7944 for the difference between the two groups, and an allocation ratio of 1:1. These values were then substituted into the sample size formula ($n=\frac{\left({q}_{1}^{-1}+{q}_{2}^{-1}\right){\left({t}_{\alpha /2}+{t}_{\beta }\right)}^{2}{\sigma }_{c}^{2}}{{\delta }^{2}}$) to determine the final estimate.

## Results

**Fig. 1:**
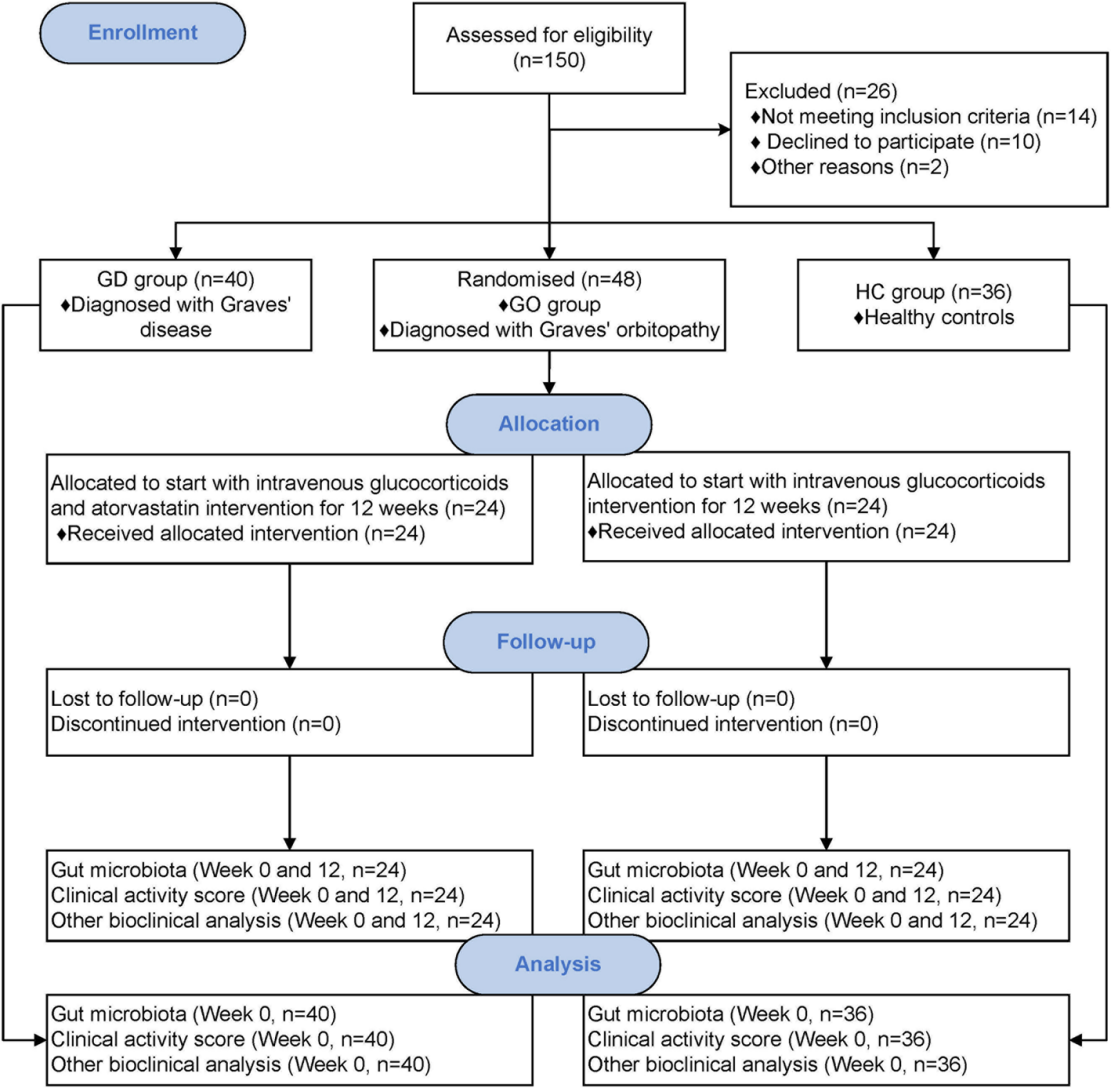
Consort flow diagram of clinical cross-sectional and randomized controlled trials

Our study first conducted a cross-sectional analysis comparing the GO, GD, and HC groups. Subsequently, the gut microbiota from these three groups was transplanted into pseudo-germ-free mice pretreated with antibiotics. Additionally, patients with GO from the case–control study received ivGCs treatment, either with or without atorvastatin. The detailed workflow of the two clinical studies is illustrated in Fig. 1.

### Case–control study baseline characteristics

In this case–control study, we enrolled 124 subjects in total, consisting of 48 patients with GO, 40 patients with GD, and 36 healthy controls (HC). In the GO group, all CAS exceeded 3 points, whereas in the GD and HC groups, all CAS were 0 points (*p* < 0.05), meeting the inclusion criteria. No statistical differences were observed in gender ratio, age, and body mass index (BMI) across the three groups (*p* > 0.05). Additionally, there were no statistically significant differences in the dosage of methimazole between the GD and GO groups (*p* > 0.05). These data demonstrating that our subjects were well-matched in terms of clinical comparability (Table 1).

**Table 1:** Patient characteristics for the case–control study

**Characteristic**	GO group (*n* = 48)	GD group (*n* = 40)	HC group (*n* = 36)	*p* value		
				*p*1	*p*2	*p*3
**Demographic**						
Age (years)	47.27 ± 11.05	45.35 ± 12.36	46.08 ± 11.18	0.732	0.438	0.641
Male [*n* (%)]	15 (31)	11 (28)	8 (22)	0.656	–	–
**Anthropometric**						
BMI (kg/m^2^)	22.44 ± 1.61	22.68 ± 2.05	21.68 ± 1.86	0.053	0.539	0.065
**Thyroid parameters**						
FT3 (pmol/L)	9.17 (5.70, 17.69)	13.48 (6.53, 34.29)	4.82 (4.43, 5.20)	** < 0.001**	0.327	** < 0.001**
FT4 (pmol/L)	27.68 (15.75, 41.51)	43.98 (21.81, 86.81)	16.24 (15.68, 17.04)	** < 0.001**	0.055	** < 0.001**
TSH (mIU/L)	0.01 (0.01, 0.02)	0.01 (0.01, 0.01)	1.71 (1.25, 2.81)	** < 0.001**	1.000	** < 0.001**
TRAb (IU/mL)	13.69 (6.88, 30.53)	7.02 (4.78, 17.54)	0.80 (0.80, 1.03)	** < 0.001**	0.203	** < 0.001**
TPOAb (IU/mL)	47.05 (2.10, 333.70)	97.17 (3.24, 885.19)	0.60 (0.40, 2.40)	** < 0.001**	0.431	** < 0.001**
TgAb (IU/mL)	0.17 (0.17, 3.05)	5.50 (0.17, 44.15)	0.17 (0.17, 1.00)	** < 0.001**	**0.012**	0.768
Duration (month)	3.5 (2.0, 6.75)	1.0 (1.0, 3.0)	–	**–**	** < 0.001**	
Methimazole (mg/day)	10.0 (7.5, 12.5)	12.5 (10.0, 15.0)	–	**–**	0.056	
CAS	5.0 (4.0, 6.0)	0.0 (0.0, 0.0)	0.0 (0.0, 0.0)	** < 0.001**	** < 0.001**	** < 0.001**

*p*1 comparison among the three groups, *p*2 GO group *vs* GD group, *p*3 GO group *vs* HC group. Continuous data comparisons among the three groups were conducted using one-way *ANOVA* or *Kruskal–Wallis* test, and post-hoc tests between two groups were conducted using *LSD t*-test or *Mann–Whitney U* test, with *Bonferroni* correction. Categorical data were analyzed using the *Chi*-square test. BMI, body mass index; FT3, free triiodothyronine; FT4, free thyroxine; TSH, thyroid stimulating hormone; TRAb, thyrotropin receptor antibody; TPOAb, thyroid peroxidase antibody; TgAb, thyroglobulin antibody; CAS, clinical activity score. Normal reference range: FT3, 3.1–6.8 pmol/L; FT4, 12.0–22.0 pmol/L; TSH, 0.27–4.2 μIU/mL; TGAb, 0–4 IU/mL; TPOAb, 0–9 IU/mL; TRAb, < 1.75 IU/L

When comparing thyroid-related clinical parameters among the three groups, we observed that, in comparison to the HC group, individuals in the GO group exhibited significantly elevated levels of FT3, FT4, TRAb, TPOAb and CAS (*p* < 0.05). This was accompanied by a notable reduction in TSH levels (*p* < 0.05). However, the TgAb levels remained unchanged (*p* > 0.05). On the other hand, when compared with the GD group, the GO group demonstrated a decrease in TgAb and CAS levels (*p* < 0.05) exclusively, with no significant variation in the remaining thyroid clinical parameters (Table 1).

### Patients with GO exhibited distinct gut microbiota profiles

**Fig. 2:**
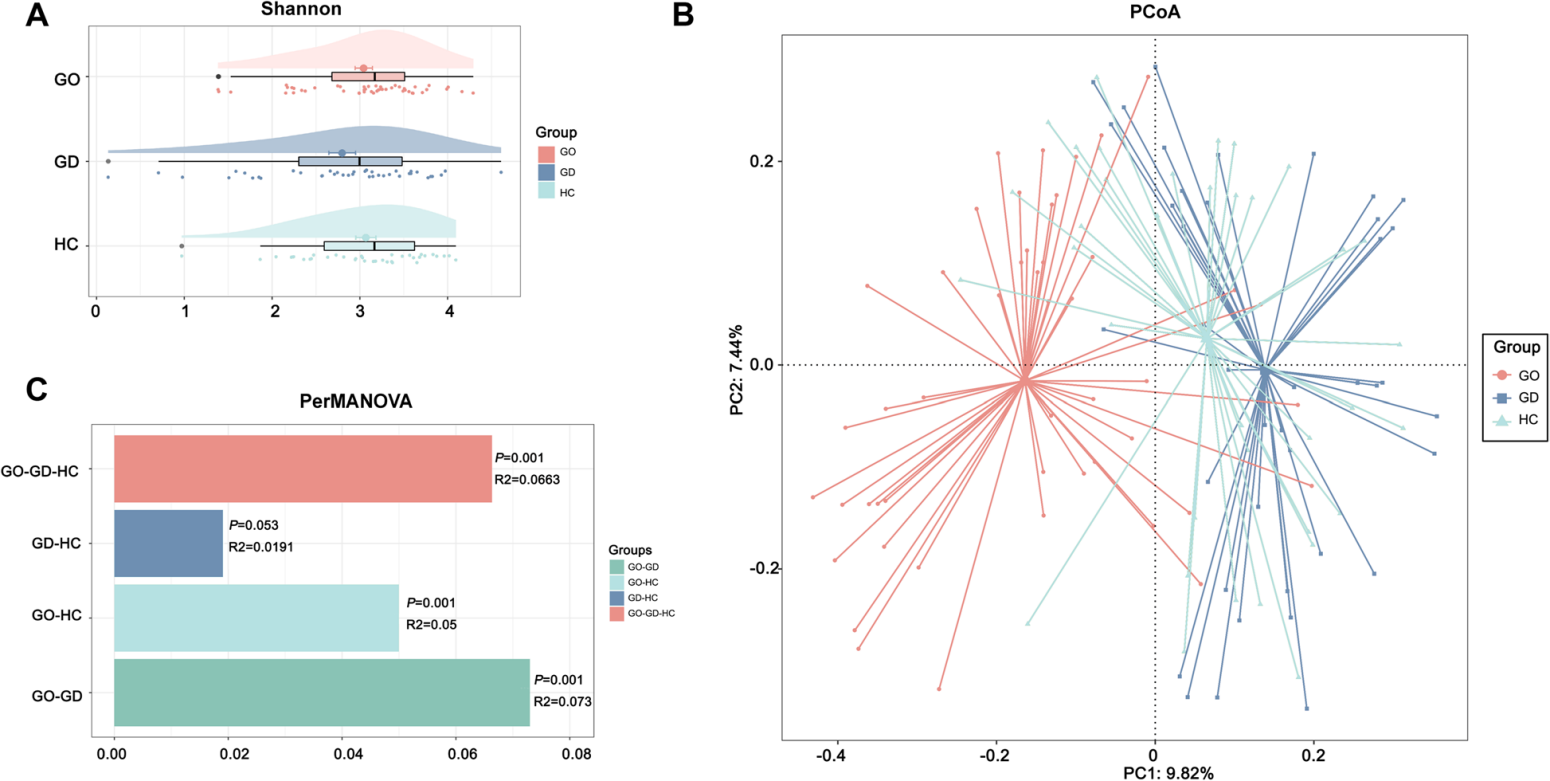
The gut microbiota's overall structure in patients with GO differs significantly from that observed in patients with GD and in HC

While we observed no significant differences in alpha diversity between the GO group and the other two groups (Fig. 2A and Supplementary Table 1), our analysis using PCoA based on Bray–Curtis dissimilarity revealed a distinct overall gut microbiome structure in the GO group compared to the GD and HC groups (Fig. 2B). To affirm the statistical significance of this divergence, we employed PerMANOVA analysis (*p* = 0.001) (Fig. 2C). Subsequently, we delved into more granular differences in the gut microbiome across the three groups.

**Fig. 3:**
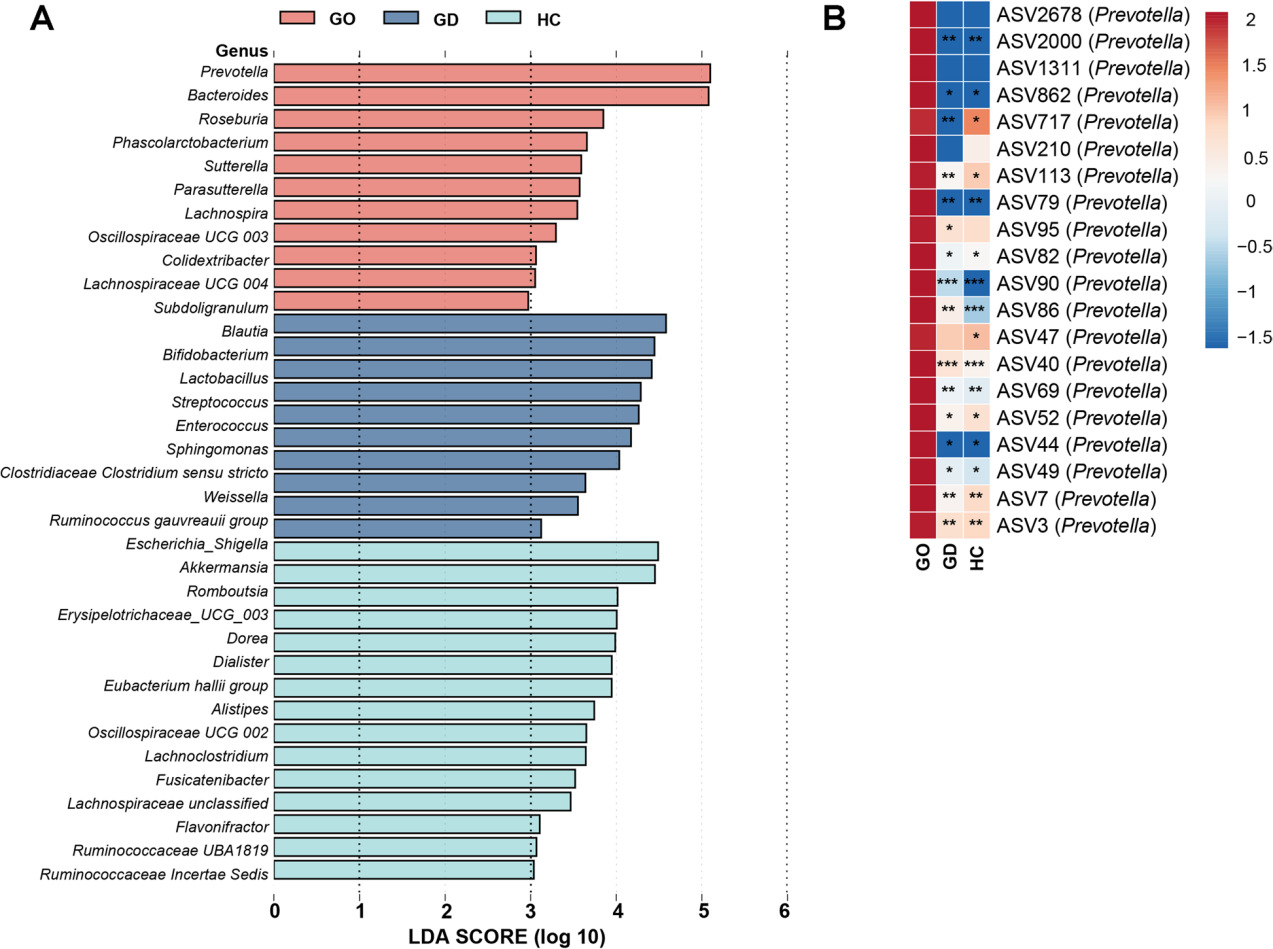
Specific differences in the gut microbiota of patients with GO, GD, and HC

Next, we utilized LEfSe analysis to pinpoint gut microbiota that have a significant impact on the stratification of samples. We identified 36 genera demonstrating notable differences across the GO, GD, and HC groups. Notably, the GO group exhibited a marked rise in the relative abundance of *Prevotella* and *Bacteroides* (Fig. 3A). To exclude the effects of age, disease course, and the use of methimazole on the LefSe analysis, we conducted a MaAsLin2 analysis. Although some gut microbiota are associated with disease course (Supplementary Fig. 1), their overlap with the differential gut microbiota identified in the LefSe analysis was minimal. Subsequently, we identified 20 ASVs belonging to the *Prevotella*, all of which showed a significant increase in the GO group (Kruskal–Wallis test, *p* < 0.05). Among these, 15 ASVs demonstrated statistical significance in post-hoc tests when compared with both the GD and HC groups (Mann–Whitney* U* test, *p* < 0.05), suggesting that these ASVs may play a role in the onset and progression of the disease in patients with GO (Fig. 3B).

**Fig. 4:**
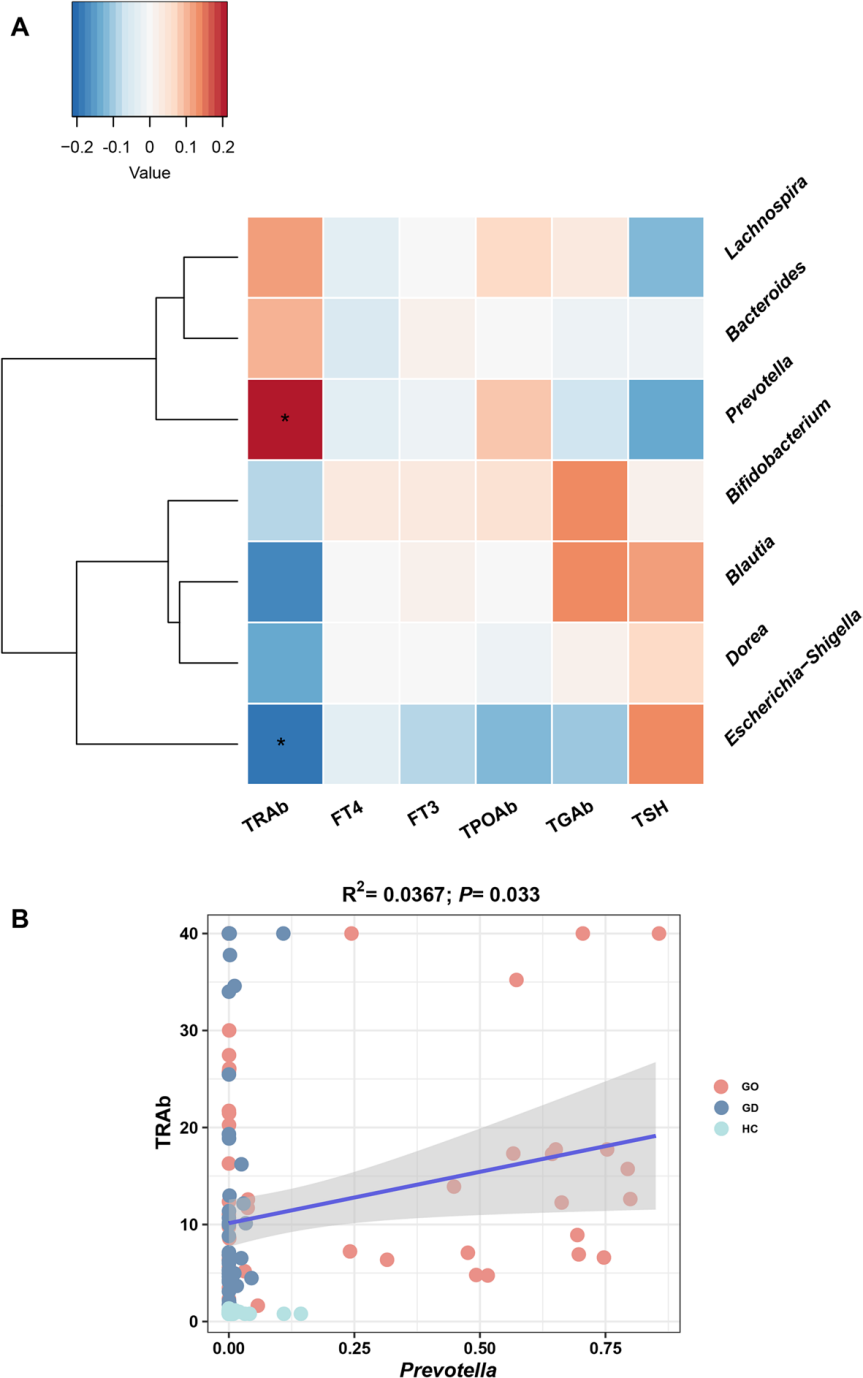
Changes in the gut microbiota were associated with thyroid-related clinical parameters

In further investigation into the connection between distinct gut microbiota compositions and the development of GO disease, we carried out a Spearman correlation analysis linking the gut microbiota with clinical parameters. Notably, *Prevotella*, markedly increased in the GO group, was found to have a significant positive correlation with TRAb, a key marker of GO (Fig. 4A). To validate this finding, we conducted a Pearson correlation analysis between *Prevotella* and TRAb, which also revealed a similar positive association (Fig. 4B).

In addition to the gut microbiota contributing to disease progression by increasing inflammatory factors, microbial translocation is also an important mechanism through which the gut microbiota influences extraintestinal diseases [22, 23]. We sequenced the ocular surface microbiota of the three groups and compared it with their gut microbiota. While the overall composition of the ocular and gut microbiota differed significantly, *Prevotella* was notably more abundant in the ocular microbiota of the GO group, followed by the GD group, with minimal presence in the HC group (Supplementary Fig. 2).

### Gut microbiota from patients with GO induced more severe intestinal barrier disruption in BALB/c mice

To explore the potential impact of dysregulated gut microbiota on the development of GO, we transplanted fecal microbiota from individuals with GO, GD, and HC into BALB/c mice, which were previously treated with broad-spectrum antibiotics. We sequenced the fecal samples from mice after the gavage period, along with the donor bacterial suspensions. Based on Bray–Curtis distances, we assessed the β-diversity of the gut microbiota in mice and their corresponding donor bacterial suspensions across the three groups. The results indicated that the gut microbiota structure of each mouse group more closely resembled that of its respective donor, suggesting successful colonization of the human-derived microbiota (Supplementary Fig. 3).

Next, we observed the weight gain of mice that received human gut microbiota transplantation. Mice receiving the microbiota from the GO group exhibited a significantly slower rate of weight gain compared to those with microbiota from the HC group (Supplementary Fig. 4).

**Fig. 5:**
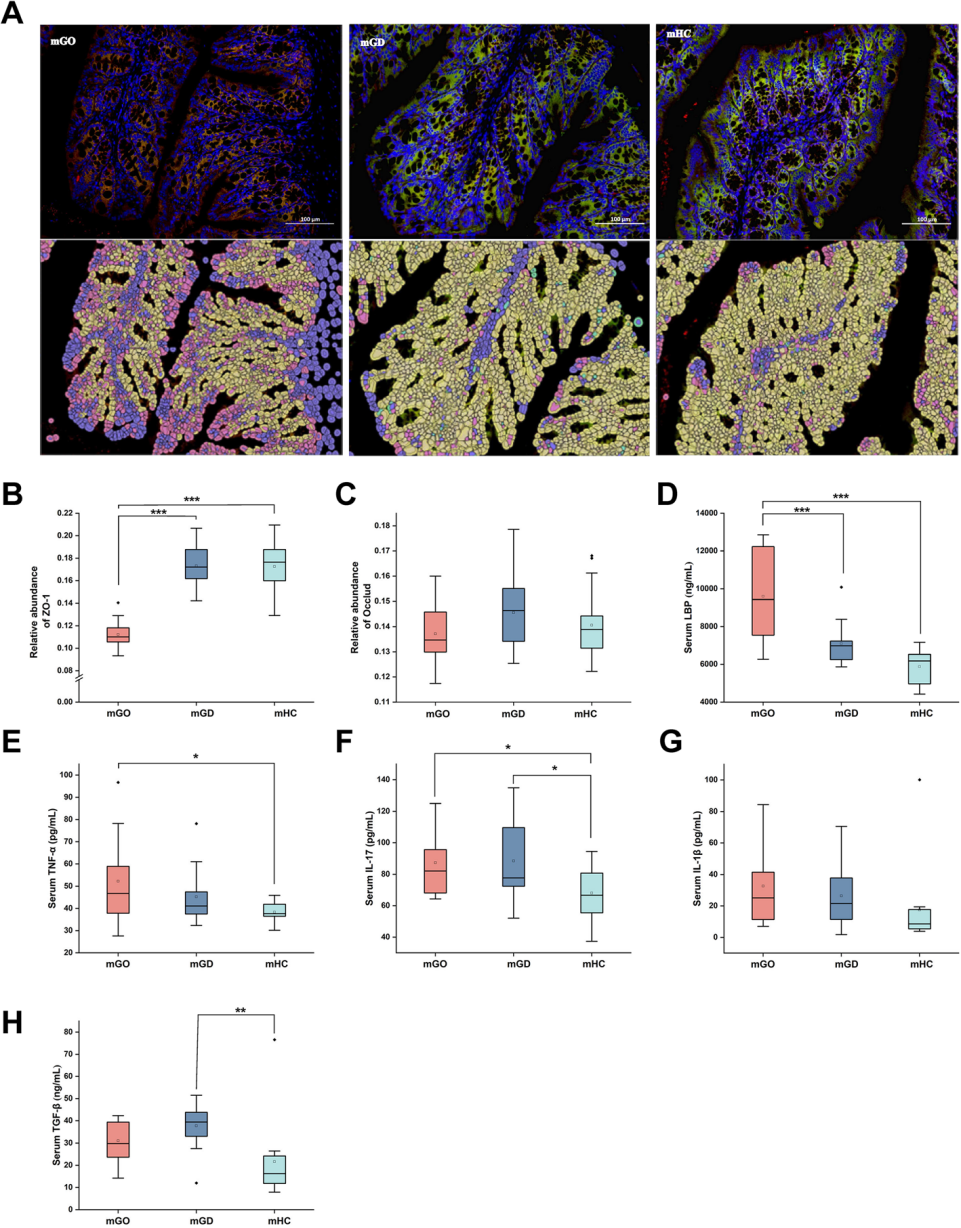
Gut microbiota transplantation from patients with GO compromised the intestinal barrier integrity in BALB/c mice

Further investigation into the integrity of the intestinal barrier was conducted through immunofluorescence analysis, focusing on the expression levels of the tight junction proteins ZO-1 and Occludin within colonic tissue samples. This analysis delineated a significant diminution in ZO-1 expression amongst mice harboring GO-derived microbiota, whereas Occludin levels remained comparably unchanged across the groups (Fig. 5A–C).

To further investigate the potential mechanisms through which gut microbiota influences disease progression, we measured inflammatory factors levels in serum and intestinal tissues. The results demonstrated that mGO mice colonized with gut microbiota from GO patients exhibited significantly elevated serum LBP levels, which can bind to antigens such as endotoxin produced by bacteria and represent a surrogate biomarker that links bacterial antigen load in the blood with host inflammatory response, along with markedly increased levels of inflammatory factors such as TNF-α (Fig. 5D–H). However, discernible differences in inflammatory profiles among the groups were not observed (Supplementary Fig. 5).

### Combining atorvastatin with ivGCs improved GO and decreased Prevotella levels

Considering the potential role of gut microbiota dysbiosis in accelerating the onset and development of GO, we seek to explore whether the gut microbiota contributes to the effectiveness of the most recent therapeutic approach for GO, which involves a combination of atorvastatin and ivGCs.

#### Atorvastatin combined with ivGCs better reduced CAS in patients with GO

We carried out a 12-week intervention study with patients with GO (*n* = 48), initially identified in our case–control research (Fig. 1). These patients were randomly allocated into two groups: one treated with a combination therapy of atorvastatin and ivGCs (ST group, *n* = 24), and the other treated with ivGCs alone (NST group, *n* = 24). Patients in both the ST and NST groups received dose-matched methimazole therapy to manage hyperthyroidism (*p* > 0.05). At baseline, our analysis revealed no significant differences between the groups regarding age, gender, BMI, lipids, thyroid parameters, and GO-related ocular symptoms. Post-intervention, however, the ST group demonstrated significantly lower CAS and TG levels compared to the NST group (*p* < 0.05), while other parameters remained comparable between groups (*p* > 0.05) (Table 2). Moreover, throughout the intervention period, 11 patients with GO (4 in the ST group and 7 in the NST group) reported adverse reactions. These included insomnia (*n* = 6), high blood pressure (*n* = 2), heart palpitations (*n* = 1), infections (*n* = 1), and abdominal discomfort (*n* = 1). Importantly, there were no severe or unforeseen incidents (Table 2).

**Table 2:** Patient characteristics for the case–control study

**Characteristic**	ST group (*n* = 24)		NST group (*n* = 24)		*P* value	
	Pre-treatment	Post-treatment	Pre-treatment	Post-treatment	*p1*	*p2*
**Demographic**						
Age (years)	47.75 ± 10.28	–	46.79 ± 11.98	–	0.767	–
Male [*n* (%)]	9 (38)	–	6 (25)	–	0.350	–
**Anthropometric**						
BMI (kg/m^2^)	22.41 ± 1.81	22.25 ± 1.36	22.47 ± 1.42	22.41 ± 1.13	0.894	0.664
**Lipids**						
TC (mmol/L)	4.37 (3.77, 4.69)	3.90 (3.58, 4.26)^***^	3.96 (3.38, 4.56)	3.73 (3.44, 4.35)	0.541	0.934
TG (mmol/L)	1.05 (0.98, 1.25)	0.97 (0.80, 1.14)^***^	1.23 (0.89, 1.53)	1.16 (0.92, 1.31) ^††^	0.404	**0.027**
HDL-C (mmol/L)	1.27 (1.11, 1.42)	1.23 (1.13, 1.33)	1.25 (1.05, 1.52)	1.25 (1.08, 1.38)	0.855	0.981
LDL-C (mmol/L)	2.36 (2.18, 2.54)	2.18 (2.05, 2.35)^**^	2.32 (1.94, 2.45)	2.11 (1.83, 2.37)^††^	0.821	0.527
**Thyroid parameters**						
FT3 (pmol/L)	9.11 (6.21, 16.77)	5.33 (4.55, 7.15)^***^	8.96 (5.18, 16.84)	5.76 (3.81, 6.49)^††^	0.773	0.536
FT4 (pmol/L)	27.37 (16.66, 38.69)	15.91 (14.31, 18.56)^***^	27.03 (15.12, 40.49)	16.25(13.23, 19.13)	0.464	0.550
TSH (mIU/L)	0.01 (0.01, 0.07)	1.11 (0.25, 2.82)^*^	0.02 (0.01, 1.20)	1.71 (0.33, 2.33)^†^	0.328	0.489
TRAb (IU/mL)	12.63 (6.56, 21.60)	3.53 (1.82, 8.92)^***^	10.32 (6.49, 21.90)	3.62 (2.53, 9.35)^††^	0.688	0.433
TPOAb (IU/mL)	60.90 (2.20, 226.35)	69. 35 (9.50, 134.50)	8.35 (1.05, 52.85)	7.76 (1.22, 99.10)	0.129	0.103
TgAb (IU/mL)	0.99 (0.17, 10.00)	0.17 (0.17, 19.53)	1.35 (0.17, 55.60)	0.86 (0.17, 9.20)^†^	0.743	0.688
Adverse reaction [*n* (%)]	–	4 (16.67)	–	7 (29.17)	–	0.308
Duration (month)	4.00 (2.00, 6.75)	–	3.00 (2.00, 6.75)	–	0.553	–
Methimazole (mg/day)	10.00 (7.5, 15.00)	–	10.0 (7.5, 10.00)	–	0.343	–
**GO-related eye symptoms**						
CAS	5.0 (4.0, 6.0)	2.0 (2.0, 3.0)^***^	5.0 (4.0, 5.0)	3.0 (3.0, 4.0)^††^	0.337	**0.012**
Changes in the value of CAS	–	3.0 (1.0, 4.0)	–	2.0 (0.0, 3.0)	–	**0.036**
Changes in the rate of CAS (%)	–	50.0 (20.0, 60.0)	–	33.3 (0.0, 45.0)	–	**0.014**
Proptosis (mm)	21.00 (19.25, 22.00)	20.00 (19.00, 20.75)^***^	21.00 (20.00, 22.00)	20.00 (20.00, 21.75)	0.849	0.162
Ocular motility restriction [*n* (%)]	14 (50)	9 (37.50)^*^	14 (50)	11 (45.83)	1.000	0.770
Diplopia [*n* (%)]	13 (54.17)	7 (29.17)^*^	15 (62.50)	11 (45.83)^†^	0.770	0.371
Intraocular pressure (mmHg)	19.00 (16.50, 24.50)	18.00 (17.25, 19.00)^**^	18.00 (16.00, 19.75)	17.00 (16.00, 19.00)^†^	0.238	0.139

*p*1, ST-pre group *vs *NST-pre group; *p*2, ST-post group *vs* NST-post group; ^*^p<0.05, ^**^p<0.01, ^***^p<0.001, ST-pre group* vs* ST-post group; ^†^*p*<0.05, ^††^*p*<0.01, ^†††^*p*<0.001, NST-pre group *vs *NST-post group. *Wilcoxon* test was used for within-group comparisons and *Mann-Whitney U *test for between-group comparisons. ST group, atorvastatin combined with ivGCs treatment; NST group, ivGCs treatment; BMI, body mass index; FT3, free triiodothyronine; FT4, free thyroxine; TSH, thyroid stimulating hormone; TRAb, thyrotropin receptor antibody; TPOAb, thyroid peroxidase antibody; TgAb, thyroglobulin antibody; ivGCs, intravenous glucocorticoids; CAS, clinical activity score. TC, total cholesterol; TG, triglyceride; HDL-C, High density lipoprotein cholesterol; LDL-C, Low density lipoprotein cholesterol. Normal reference ranges: FT3, 3.1-6.8 pmol/L, FT4, 12.0-22.0 pmol/L, TSH, 0.27-4.2 μIU/mL, TGAb, 0-4 IU/mL, TPOAb, 0-9 IU/mL, TRAb, <1.75 IU/L, TC, 2.33-5.17 mmol/L, TG, 0-1.7 mmol/L, HDL-C, 1.2-1.68 mmol/L, LDL-C, 1.9-3.12 mmol/L

**Fig. 6:**
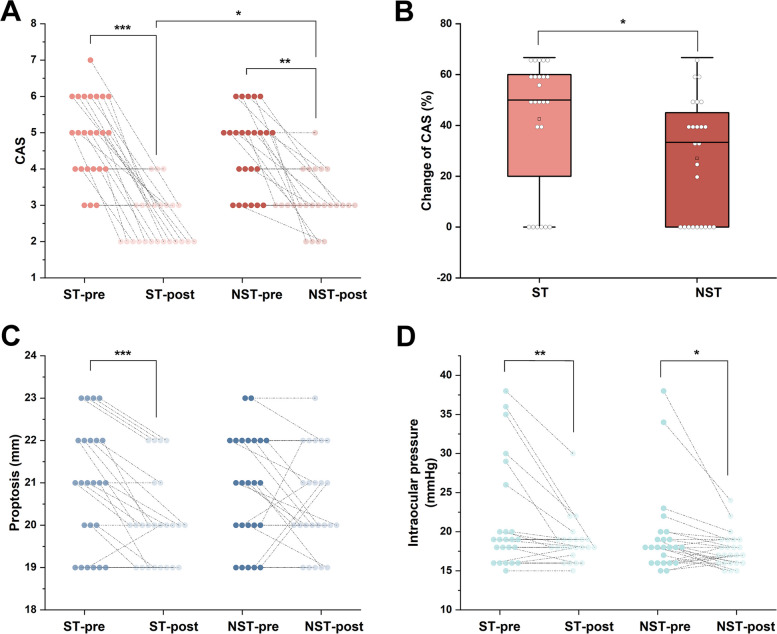
The comparison of GO-related ocular symptoms before and after intervention in the ST and NST groups

Subsequently, we assessed the variations in our primary observational indicator, the CAS, which was not different between the two groups at baseline, and found substantial improvements in CAS for both groups following the intervention (Fig. 6A and Table 2). Notably, ST group exhibited lower CAS levels (Fig. 6A and Table 2), indicating enhanced therapeutic efficacy. Furthermore, the rate of change in CAS was significantly higher in ST group compared to NST group (Fig. 6B and Table 2). There were 6 patients receiving combination therapy and 9 patients receiving monotherapy whose CAS did not improve post-intervention (Fig. 6B and Table 2). Additionally, the ST group exhibited significant post-intervention improvements in both proptosis and intraocular pressure (Fig. 6C, Table 2). While the NST group also showed significant reduction in intraocular pressure (Fig. 6D, Table 2).

#### Atorvastatin combined with ivGCs promoted a healthier gut microbiota composition and induced the reduction in Prevotella

We examined the variations in gut microbiota before and after administering a treatment regimen combining atorvastatin with ivGCs, compared to a regimen of ivGCs alone. The baseline of microbiota between the two groups had no difference before intervention (Supplementary Fig. 6). Moreover, whether combined with atorvastatin or not, we did not observe significant changes in the gut microbiota’s α- and β-diversity after intervention, underscoring the gut microbiome's inherent stability (Supplementary Fig. 7 and Supplementary Tables 2, 3).

**Fig. 7:**
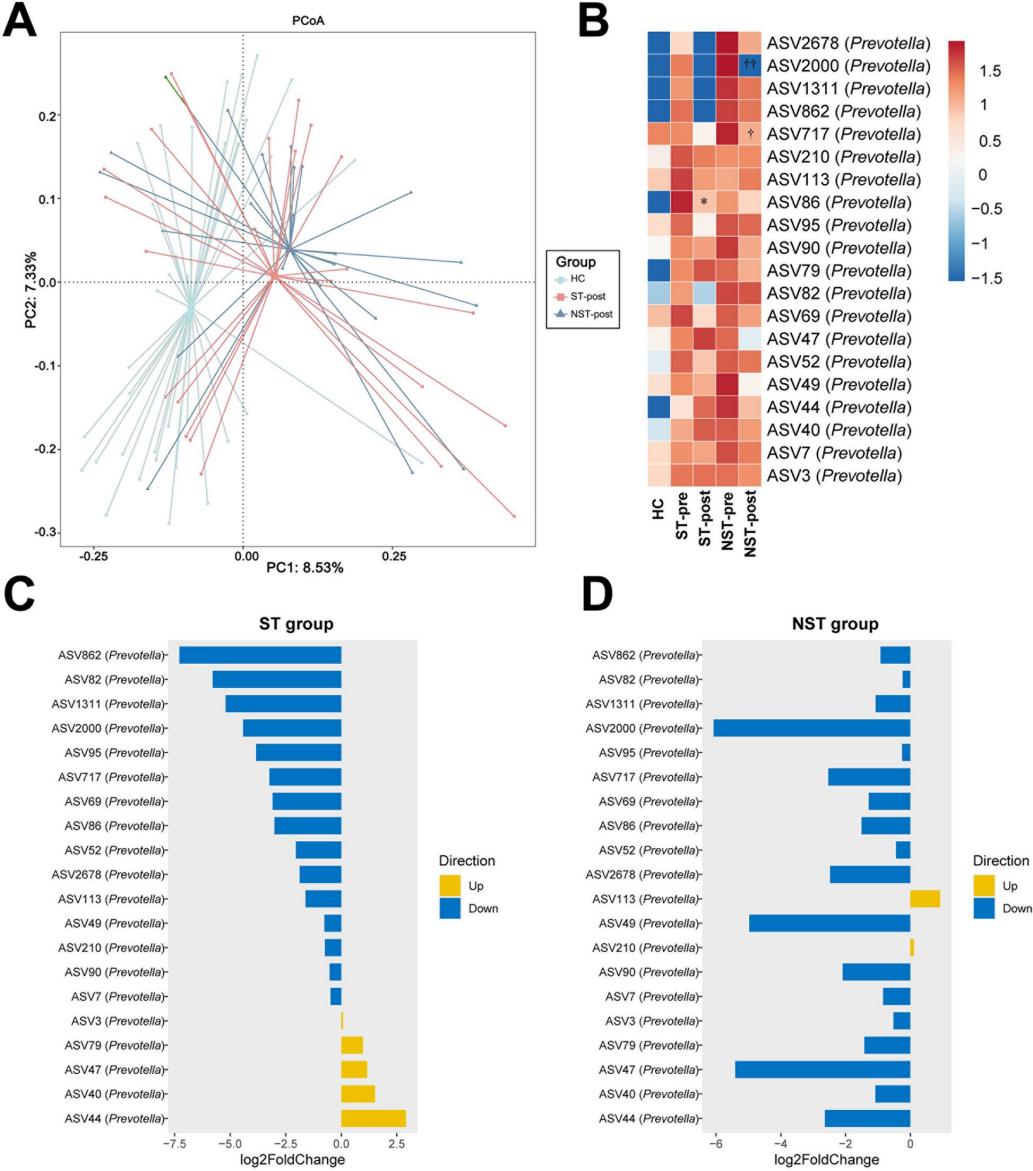
Combining atorvastatin with ivGCs brought the gut microbiota of patients with GO closer to that of healthy individuals and lowered the relative abundance of *Prevotella*

Interestingly, when we compared the gut microbiota of post-treatment patients with healthy individuals from our previous case–control study, we found that adding atorvastatin to ivGCs treatment brought the gut microbiota of patients with GO closer to healthy levels. This is evident from the PCoA analysis based on Bray–Curtis distance, where the gut microbiota of patients with GO receiving combined atorvastatin and ivGCs treatment was more similar to that of healthy individuals than those treated with ivGCs alone (Fig. 7A).

Further analysis was conducted on 20 crucial ASVs (belonging to the *Prevotella*) identified through our case–control study, aiming to assess the potential effects of these therapeutic interventions on specific ASVs. The combined treatment regimen of atorvastatin and ivGCs was observed to significantly reduce majority relative abundance of these ASVs (Fig. 7B, C). Similarly, the administration of ivGCs alone was also effective in diminishing the levels of key ASVs (Fig. 7B, D), thereby suggesting a discernible impact of these treatments on the gut microbiota’s composition.

## Discussion

The role of gut microbiota in the etiology of GO has been previously suggested [8, 24–26]. Nonetheless, clinical research on the relationship between gut microbiota and GO remains limited, with most studies being cross-sectional rather than prospective interventions aimed at understanding the microbiota's role in GO treatment [9, 10].

We show in a case–control study that patients with GO revealed a unique composition of gut microbiota, marked by significant increases in the *Prevotella* and *Bacteroides* genera, with *Prevotella* notably correlating positively with TRAb levels. When the gut microbiota from patients with GO—rather than those with GD or healthy individuals—were transferred into BALB/c mice that had been pre-treated with an antibiotic cocktail, the mice experienced enhanced intestinal barrier dysfunction, accompanied by a significant increase in serum LBP and inflammatory factor levels, as well as delayed weight gain. Moreover, the adoption of a novel therapeutic strategy for GO, which combines atorvastatin with ivGCs [11, 27], not only markedly improved the ocular clinical symptoms (such as CAS) in patients but also fostered a healthier gut microbiota composition, leading to a notable reduction in the relative abundance of *Prevotella*. These findings highlight the potential pivotal role that gut microbiota play in the progression of GO, while also providing some clinical validation for the association between statin treatment and a reduced incidence of gut microbiota dysbiosis.

GO is the primary extrathyroidal manifestation of GD; however, not all patients with GD exhibit GO [1]. Previous studies have found that patients with GD have an imbalance in their gut microbiota, mainly characterized by a decrease in the diversity of the gut microbiota [28] and an increase in genus such as *Lactobacillus* and *Veillonella* [24]. Our previous study further found that the changes in the gut microbiota of patients with GD are related to immune dysregulation [29]. We recruited patients with GO and GD with well-matched thyroid-related clinical parameters, along with a healthy control group, and found that the gut microbiota composition in patients with GO differed significantly from that in patients with GD and healthy individuals, suggesting its potential role in the progression of GO.

A comparative cross-sectional study of the gut microbiota in 33 patients with GO and 32 healthy individuals found significantly higher levels of *Bacteroides* and *Prevotella* genera in patients with GO [30]. Similarly, another study focusing exclusively on patients with GO echoed these findings, additionally highlighting a positive correlation between the *Prevotella* genus and TRAb levels [31]. Our results align with these previous findings. However, the recent INDIGO multicenter European study, while also recognizing *Bacteroides* as a biomarker for GO, reported a decrease in its relative abundance in patients with GO [10]. This was paralleled by observations in their ddH_2_O-TSHR mice model [8]. Reflecting the concept that genetic predisposition to GD varies with geography and ethnicity [32], this variation in microbial abundance may also be influenced by such factors. Notably, the subjects in both our and the aforementioned studies were of Asian descent, in contrast to the European cohort of the INDIGO study.

Extensive research has demonstrated a significant relationship between TRAb and the activity of GO, establishing TRAb as a predictive marker for the severity and progression of GO [33–35]. It is now recognized as a key biomarker for this condition [1, 36]. Our investigation, in conjunction with the work conducted by Shi et al. [31], identified a notable positive correlation between TRAb levels and the presence of the *Prevotella*. This finding suggests a potential role of *Prevotella* in contributing to the immune dysregulation observed in GO. In addition, the comparable TRAb expression levels in both our GO and GD groups lend additional support to the critical involvement of *Prevotella* in the pathology of GO disease. An elevated presence of *Prevotella* has been documented in a range of other immune-mediated conditions, including rheumatoid arthritis [37–40], psoriasis [41], systemic lupus erythematosus [42], and Duchenne muscular dystrophy [43], indicating its broader impact on immune health [44]. Furthermore, researchers have found that *Prevotella intermedia* in the oral cavity can exacerbate subclinical hypothyroidism [45]. Based on the above studies and our case–control study findings, we hypothesize that gut microbiota dysbiosis, particularly the increased abundance of *Prevotella*, may play a role in the immune dysregulation of GO and contribute to its development and progression.

LPS, a key component of gram-negative bacterial outer membranes, is highly immunogenic and pro-inflammatory. TNF-α and IL-17 have been identified as key inflammatory factors in the development and progression of GO [46]. In our animal study exploring the potential causal relationship between gut dysbiosis and GO, we found that mice receiving gut microbiota from patients with GO showed significant intestinal barrier damage, facilitating the systemic spread of LPS and elevating inflammatory factors such as TNF-α and IL-17. Moreover, these mice experienced slower weight gain. Furthermore, Moshkelgosha et al. found that vancomycin-induced depletion of gut microbiota in ddH_2_O-TSHR mice significantly slowed the progression of GD/GO, providing further evidence for a causal relationship between gut microbiota and GO [8].

Several studies have found that specific antibodies of the *Prevotella* mediated pro-inflammatory Th-17/Th-1 immune responses, breaking down the intestinal mucosal barrier and leading to the systemic spread of inflammatory mediators, bacteria, and bacterial products [47]. This also explains the observation in our animal experiments. We speculate that the dysbiosis gut microbiota in patients with GO, including an increase in *Prevotella*, may trigger the occurrence of “leaky gut” and subsequent bacterial endotoxin dissemination, leading to pro-inflammatory Th-17/Th-1 immune responses, promoting the release of IL-17, and activating the NF-κB signaling pathway, resulting in increased TNF-α levels [48]. Meanwhile, enhanced TNF-α/TNFR pathway activity may also promote Th17 differentiation and IL-17 production, further contributing to the development of GO [49]. Interestingly, although we did not analyze the orbital tissue of the mice, our analysis of the ocular surface microbiota in patients with GO revealed a significant increase in *Prevotella*, suggesting a possible microbial shift. These speculations, however, require further validation in subsequent studies.

Increasing evidence indicates that the efficacy of various pharmaceuticals is intricately linked to the diverse functionalities within the gut microbiome [13]. According to a population-based cross-sectional study, statin usage is correlated with reduced gut dysbiosis, including *Prevotella* modulation [13]. The gut microbiome’s composition also plays a crucial role in modulating the host's response to statin medications [14]. In our intervention study, we corroborated the findings of Lanzolla et al.’s phase II clinical trial for STAGO [10] that incorporating atorvastatin into ivGCs treatments markedly improved the CAS for patients suffering from GO. Concurrently, a notable reduction in *Prevotella* levels was observed. Nevertheless, regardless of whether atorvastatin was added or not, treatment with ivGCs alone led to a statistically significant decrease in specific ASVs of the *Prevotella*. This observation aligns with expectations since high-dose glucocorticoid therapy in rheumatoid arthritis patients is known to diminish *Prevotella* levels [39]. However, it is undeniable that incorporating atorvastatin into ivGCs treatment significantly aligns the gut microbiota composition of patients with GO closer to that of healthy individuals. Interestingly, although atorvastatin is known to reduce TC and LDL-C levels, our study found no significant changes in TC and LDL-C levels either between or within groups, both before and after intervention. This may be due to the relatively low baseline TC and LDL-C levels in our enrolled patients. Furthermore, this finding suggests that atorvastatin may exert its protective effects on patients with GO through mechanisms mediated by the gut microbiota rather than by lowering TC and LDL-C.

However, we recognize some limitations within our study. First, the sample size of our clinical investigation is limited, which highlights the need for more extensive, prospective, and interventional research on larger human cohorts in the future. But our study’s findings can act as a foundational dataset for future, expansive cohort studies. Second, despite observing a correlation between the *Prevotella* and TRAb, we have not yet pinpointed the exact bacterial strains responsible for the initiation and progression of GO. We plan to delve deeper into this matter, guided by our microbiome sequencing outcomes. Moreover, the intricacies of the gut microbiome’s molecular role in GO’s development remain elusive, necessitating further investigation.

## Conclusion

In conclusion, for patients with GO, an imbalance in the gut microbiome, particularly concerning *Prevotella* levels, may play a critical role in the disease’s progression. The combination therapy of atorvastatin and ivGCs might slow the progression of GO by reducing *Prevotella* abundance. Our findings suggest that closer attention to the interactions between medications, the host, and the gut microbiota is warranted to potentially delay the progression of GO.

## Supplementary Information

Additional file 1: Supplementary Fig. 1. The MaAsLin2 analysis revealed that the gut microbiome at the genus level is associated with disease course. Supplementary Fig. 1. The MaAsLin2 analysis revealed that the gut microbiome at the genus level is associated with disease course. Supplementary Fig. 3. Bray-Curtis distance between the mouse and donor in each group. Supplementary Fig. 4. Changes in the weight of BALB/c mice before and after fecal microbiota transplantation. Supplementary Fig. 5. Comparison of inflammation factor levels in three groups of mice after fecal microbiota transplantation. Supplementary Fig.6. The overall structure of the gut microbiota between ST and NST groups before the intervention. Supplementary Fig. 7. The overall structure of the gut microbiota before and after intervention.

Additional file 2: Supplementary Table 1. Comparison of alpha diversity among GO, GD and HC groups. Supplementary Table 2. Comparison of alpha diversity pre- and post- atorvastatin combined with ivGCs treatment. Supplementary Table 3. Comparison of alpha diversity pre- and post- ivGCs treatment.

## Data Availability

The sequencing data of the gut microbiota from this study can be accessed from the NCBI BioProject repository (https://www.ncbi.nlm.nih.gov/, accession number PRJNA1138456). The temporary link before the data release is: https://dataview.ncbi.nlm.nih.gov/object/PRJNA1138456?reviewer=6g885hccrr48435ennt07g107d.
