# Supplementary material for: Modulation of gut microbiota in Graves’ orbitopathy: Prevotella dominance and atorvastatin’s impact
Source: Microbiome. 2025 Dec 29;13:258. doi: 10.1186/s40168-025-02219-2 (PMC12751797; doi:10.1186/s40168-025-02219-2)
Supplement: Supplementary file 2 — Additional file 1: Supplementary Fig. 1. The MaAsLin2 analysis revealed that the gut microbiome at the genus level is associated with disease course. Supplementary Fig. 1. The MaAsLin2 analysis revealed that the gut microbiome at the genus level is associated with disease course. Supplementary Fig. 3. Bray-Curtis distance between the mouse and donor in each group. Supplementary Fig. 4. Changes in the weight of BALB/c mice before and after fecal microbiota transplantation. Supplementary Fig. 5. Comparison of inflammation factor levels in three groups of mice after fecal microbiota transplantation. Supplementary Fig.6. The overall structure of the gut microbiota between ST and NST groups before the intervention. Supplementary Fig. 7. The overall structure of the gut microbiota before and after intervention. [file 40168_2025_2219_MOESM1_ESM.docx]

**Supplementary Fig.1** The MaAsLin2 analysis revealed that the gut microbiome at the genus level is associated with disease course.





**Supplementary Fig. 2** The composition of ocular microbiota in the GO, GD, and HC groups and its relationship with the gut microbiota.

**A** Heatmap showed the differential gut microbiota identified in the GO group and its presence in the ocular surface microbiota across the GO, GD, and HC groups. Red indicates higher relative abundance, while blue indicates lower.

**B** LefSe analysis identified 37 differentially abundant ocular surface microbiota genera among the three groups. The red, blue, and green histograms indicate genera that were enriched in the GO, GD, and HC groups, respectively.

GO, Graves' orbitopathy; GD, Graves' disease; HC, healthy control; LEfSe, linear discriminant analysis effect size.


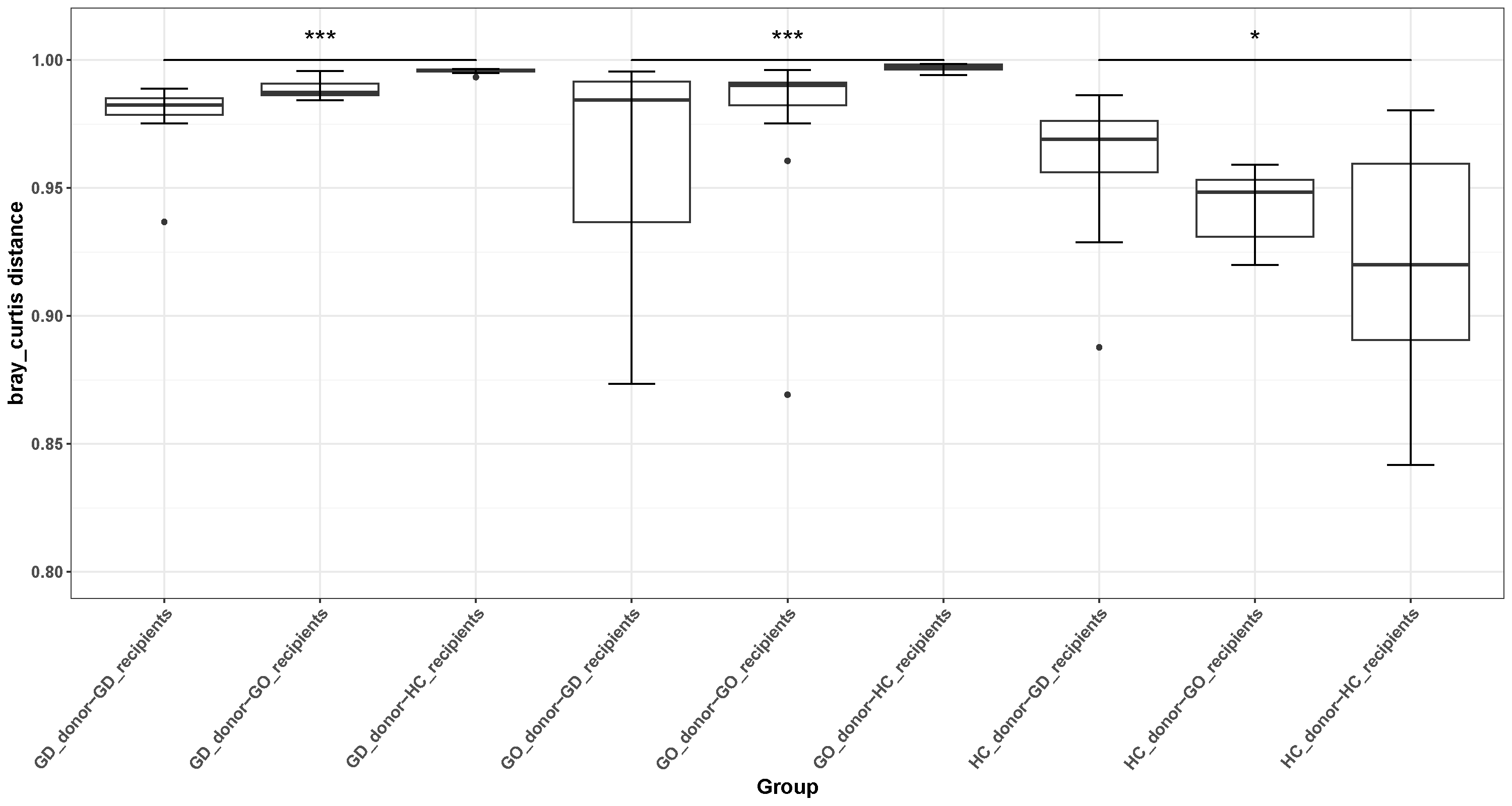


**Supplementary Fig. 3** Bray-Curtis distance between the mouse and donor in each group.

The comparison among the three groups was conducted using the *Kruskal-Wallis* test. ^*^*p*<0.05, ^**^*p*<0.01, ^***^*p*<0.001. GO, Graves' orbitopathy; GD, Graves' disease; HC, healthy control (n=5 per group).





**Supplementary Fig. 4** Changes in the weight of BALB/c mice before and after fecal microbiota transplantation.

The comparison among the three groups was conducted using the *Kruskal-Wallis* test, and the post-hoc comparison between two groups was performed using the *Mann-Whitney U* test, with *Bonferroni* correction. ^*^*p*<0.05. The mGO, mGD and mHC groups refer to mice that received fecal microbiota transplants from human with Graves' orbitopathy, Graves' disease, and healthy controls, respectively. BMI, body mass index; FMT, fecal microbiota transplantation.





**Supplementary Fig. 5** Comparison of inflammation factor levels in three groups of mice after fecal microbiota transplantation.

The mGO, mGD and mHC groups refer to mice that received fecal microbiota transplants from human with Graves' orbitopathy, Graves' disease, and healthy controls, respectively. IL-1β, interleukin-1β; IL-6, interleukin-6; IL-17, interleukin-17; TGF-β, transforming growth factor-β; TNF-α, tumor necrosis factor-α.


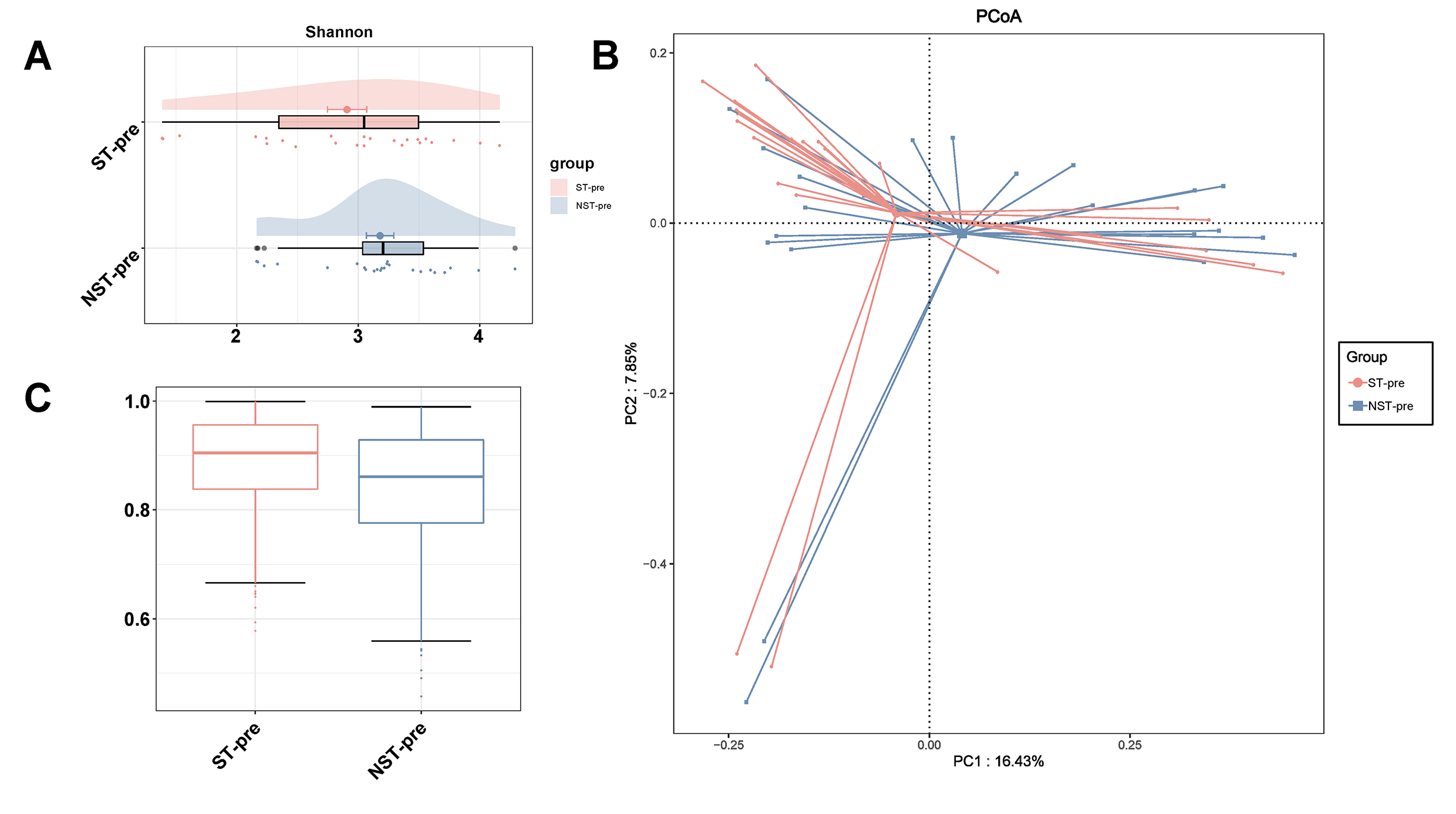


**Supplementary Fig. 6** The overall structure of the gut microbiota between ST and NST groups before the intervention.

**A** No difference in the α-diversity of the gut microbiota was observed at baseline between the ST and NST groups.

**B** PCoA based on Bray-Curtis distance could not distinguish the gut microbiota between the ST and NST groups at baseline.

**C** The *Mann-Whitney U* test suggested that there was no statistical difference between the two groups based on the Bray-Curtis distance at baseline.

ST group, atorvastatin combined with ivGCs treatment; NST group, ivGCs treatment. PCoA, principal coordinates analysis.


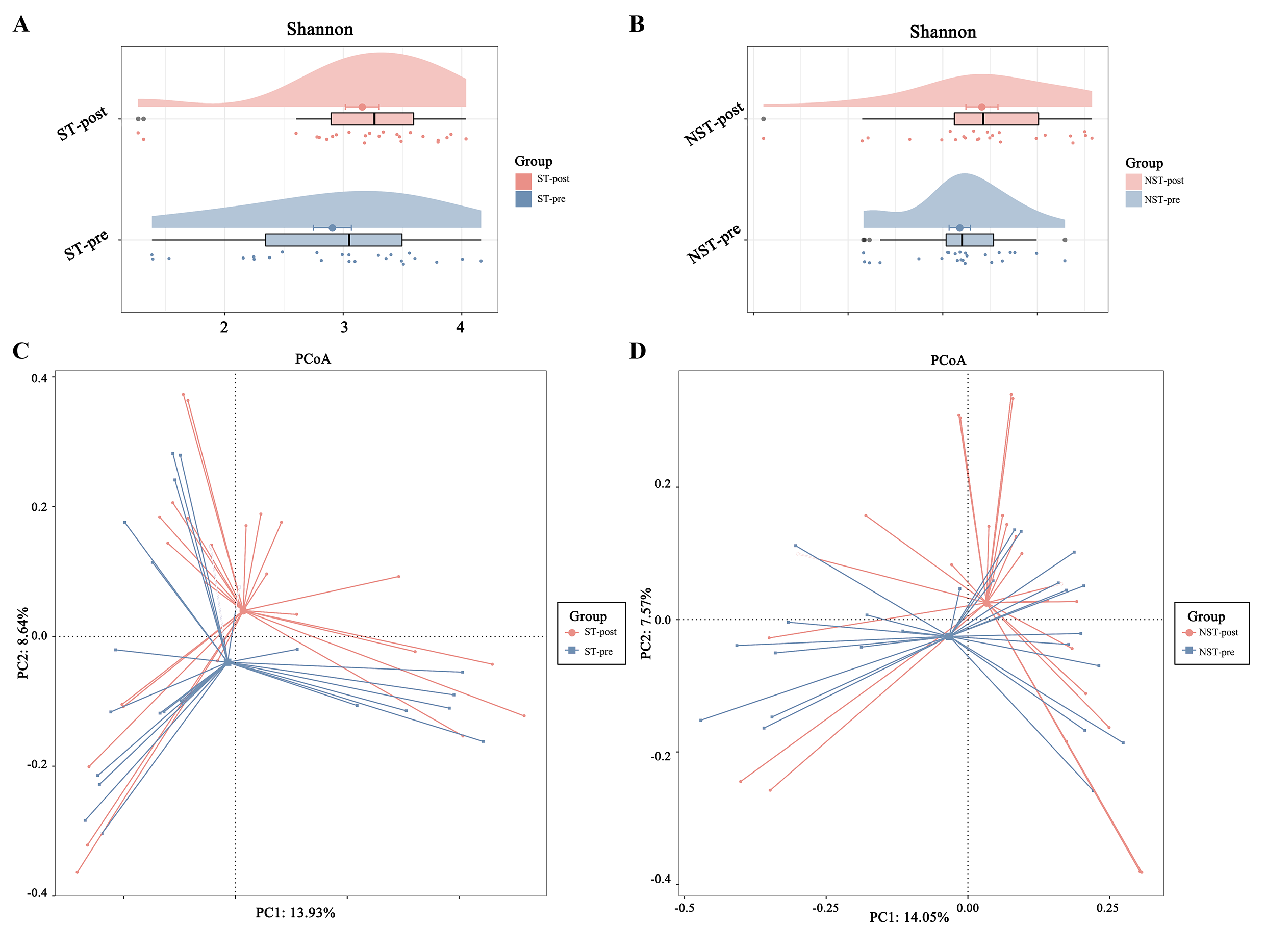


**Supplementary Fig. 7** The overall structure of the gut microbiota before and after intervention.

**A, B** No difference was observed in the alpha diversity of the gut microbiota pre- and post-treatment in the ST group **A** and NST group **B**.

**C, D** PCoA based on bray-curtis distance could not distinguished the gut microbiota of pre- and post-treatment in the ST group **C** and NST group **D**.

ST group, atorvastatin combined with ivGCs treatment; NST group, ivGCs treatment. PCoA, principal coordinates analysis.
