# Supplementary material for: Modulation of gut microbiota in Graves’ orbitopathy: Prevotella dominance and atorvastatin’s impact
Source: Microbiome. 2025 Dec 29;13:258. doi: 10.1186/s40168-025-02219-2 (PMC12751797; doi:10.1186/s40168-025-02219-2)
Supplement: Supplementary file 3 — Additional file 2: Supplementary Table 1. Comparison of alpha diversity among GO, GD and HC groups. Supplementary Table 2. Comparison of alpha diversity pre- and post- atorvastatin combined with ivGCs treatment. Supplementary Table 3. Comparison of alpha diversity pre- and post- ivGCs treatment. [file 40168_2025_2219_MOESM2_ESM.docx]

**Supplementary Table 1** Comparison of alpha diversity among GO, GD and HC groups

| Index | GO group (n=48) | GD group (n=40) | HC group (n=36) | *P* value |
| --- | --- | --- | --- | --- |
| ACE | 98.68 (82.55, 137.75) | 91.50 (64.50, 129.89) | 105.00 (78.96, 132.17) | 0.457 |
| Chao | 98.50 (82.50, 137.75) | 91.50 (64.50, 129.75) | 105.00 (78.75, 132.00) | 0.456 |
| Shannon | 3.17 (2.68, 3.51) | 3.00 (2.31, 3.48) | 3.17 (2.59, 3.62) | 0.442 |
| Simpson | 0.09 (0.06, 0.15) | 0.09 (0.07, 0.19) | 0.09 (0.06, 0.17) | 0.616 |

GO, Graves' orbitopathy; GD, Graves' disease; HC, healthy control.

**Supplementary Table 2** Comparison of alpha diversity pre- and post- atorvastatin combined with ivGCs treatment

| Index | Pre- (n=24) | Post- (n=24) | *P* value |
| --- | --- | --- | --- |
| ACE | 114.08 (78.75, 135.42) | 88.16 (69.72, 110.00) | 0.146 |
| Chao | 114.00 (78.75, 135.38) | 88.00 (69.50, 110.00) | 0.149 |
| Shannon | 0.08 (0.06, 0.11) | 0.09 (0.06, 0.19) | 0.264 |
| Simpson | 0.08 (0.06, 0.11) | 0.09 (0.06, 0.19) | 0.351 |

GO, Graves' orbitopathy; GD, Graves' disease; HC, healthy control.

**Supplementary Table 3** Comparison of alpha diversity pre- and post- ivGCs treatment

| Index | Pre- (n=24) | Post- (n=24) | *P* value |
| --- | --- | --- | --- |
| ACE | 123.25 (101.50, 163.75) | 118.00 (93.75, 147.30) | 0.364 |
| Chao | 123.00 (101.50, 163.75) | 118.00 (93.75, 147.25) | 0.375 |
| Shannon | 3.43 (3.12, 4.01) | 3.20 (3.04, 3.54) | 0.153 |
| Simpson | 0.07 (0.04, 0.09) | 0.10 (0.07, 0.13) | 0.089 |

GO, Graves' orbitopathy; GD, Graves' disease; HC, healthy control.
